# Specialty grand challenge in adrenal endocrinology

**DOI:** 10.3389/fendo.2023.1237733

**Published:** 2023-07-06

**Authors:** Henrik Falhammar

**Affiliations:** ^1^ Department of Endocrinology, Karolinska University Hospital, Stockholm, Sweden; ^2^ Department of Molecular Medicine and Surgery, Karolinska Intitutet, Stockholm, Sweden

**Keywords:** primary adrenal insufficiency (PAI), congenital adrenal hyperplasia, adrenal incidentaloma (AI), adrenocortical adenocarcinoma, pheochoromocytoma, primary aldosteronism (PA)

## Introduction

The adrenal glands are on top of each kidney and weight around 4-6 grams each in an adult person, however, they can grow 50% during stress or pregnancy. The hormones from the adrenal glands, i.e., cortisol, aldosterone, adrenal androgens and catecholamines are involved in most of the body systems. Without cortisol, e.g., we would not be able to survive (1). If tumors or hyperplasia arise from the adrenal gland any of these hormones can be produced in excess and give rise to disorders such as pheochromocytoma, primary aldosteronism and Cushing syndrome. Some conditions are common (e.g., adrenal incidentalomas) while others are rare (e.g., adrenal medullary hyperplasia). However, one common feature is that all adrenal disorders are relatively unknown to physicians and most patients have never heard of the adrenal glands. In the following few paragraphs a few examples of adrenal disorders and examples of challenges as well as new findings will be presented.

## Primary adrenal insufficiency

The most common form of primary adrenal insufficiency (PAI) in adults from high-income countries is autoimmune adrenalitis while in children it is congenital adrenal hyperplasia (CAH) (2). Another cause of PAI, adrenal tuberculosis, is claimed to be common in low- to middle income countries but very little is known of tuberculosis-induced PAI in high-income setting (3, 4). Novel forms of PAI have emerged such as immune checkpoint inhibitor (ICI) induced PAI, even though ICIs more commonly induce secondary AI (5). Bilateral adrenal metastasis and bilateral adrenal hemorrhage may result in PAI (6, 7). It is estimated that 10-20/100,000 of the population have PAI (2). Long term negative outcomes have been the focus lately, especially complications due to unphysiological glucocorticoid replacement but also adrenal crisis (8, 9). New treatments have been introduced such as modified release glucocorticoids and hydrocortisone administered subcutaneously via pump (8). On the horizon cell-based therapies emerge such as allogeneic adrenocortical cell transplantation and adrenal-like steroidogenic cell manufacturing from either stem cells or lineage conversion of differentiated cells (10).

## Congenital adrenal hyperplasia

CAH is considered a rare group of disorders affecting the steroid synthesis of which 21-hydroxylase deficiency is by far the most frequent (11, 12). CAH is one of many genetic disorders of the adrenal glands causing PAI and the most common. The incidence of classic CAH is around 1/15000 according to neonatal screening programs (11). Non-classic CAH does not have apparent cortisol insufficiency but is usually diagnosed due to adrenal androgen excess or due to family screening. The prevalence of non-classic CAH in the general US Caucasian population has been claimed to be 1/200 (13), while in a country such as Sweden with around 10 million inhabitants only 90 cases of non-classic CAH have been diagnosed (14). However, it can be assumed that most patients with non-classic CAH probably never are diagnosed (15). The prevalence needs to be studied in larger cohorts which have been screened for non-classic CAH to be able to have more well-founded data. Moreover, long-term outcome data have emerged over the last few decades (16–18). Though, most studied patients with CAH have been younger than 30 years of age, and only a few above 50 years of age, the age where most long-term outcomes can be expected to appear. Since the introduction of glucocorticoids and mineralocorticoids in the 1950s, no major advancement has been seen until recently when new therapies have begun to emerge such as modified release glucocorticoids, hydrocortisone administered subcutaneously via pump, the CYP17A1 inhibitor abiraterone, corticotropin-releasing hormone-receptor 1 antagonists, corticotropin antibodies, corticotropin receptor (melanocortin 2 receptor [MC2R]) antagonists, as well as gene- and cell-based therapies (11, 19–21).

## Adrenal incidentalomas

Adrenal incidentalomas are found in approximately 2% of the adult population and pose a rising challenge for endocrinologists worldwide demanding ever increasing resources to manage (22). Even if overt Cushing syndrome is not present, autonomous cortisol secretion (ACS) seems to be associated with increased mortality (23–25), especially in women below 65 years of age (24). There are even indications that patients with non-functional adrenal tumors have increased mortality (26). Performing adrenalectomy in patients with ACS is controversial (27), but small randomized controlled trials (RCTs) have just begun to be published (28). Moreover, there are many rare forms of adrenal incidentalomas such as adrenal myelolipomas, adrenal cysts and other adrenal lesions that require more studies to improve our understanding and management of these masses (29–31).

## Adrenal malignancy

Both adrenocortical cancer (ACC) and adrenal metastasis have poor prognosis (32, 33). Very few RCTs have been done so most treatment recommendations are based on retrospective studies or small clinical trials (34). However, since only 1 new cases of ACC are diagnosed per million, per year (35), large multinational collaborations are required to find new treatments but also to find new genetic and molecular markers.

## Pheochromocytoma and paraganglioma

The symptoms and signs of pheochromocytomas and paragangliomas (PPGLs) are diverse and can easily be misinterpreted (36). Even if only pheochromocytomas are found in the adrenal glands, while paragangliomas are found anywhere in the human body, both conditions are usually grouped since both result in catecholamine excess (37). Cardiovascular manifestations of PPGLs can be dramatic and fatal (38). Nowadays most pheochromocytomas are found in the work up of adrenal incidentalomas (36, 39), and the proportion of pheochromocytomas that are found during yearly surveillance of a genetic syndrome increase. Phenoxybenzamine, a non-selective irreversible alpha-adrenoceptor antagonist, has been the standard preoperative treatment but its use has decreased in favor of selective reversible alpha-adrenoceptor antagonists such a doxazosin which are more readily available (40). Some patients use a calcium channel blocker instead or no medication at all preoperatively which is controversial, however, large RCTs are needed to tease out the best preoperative treatment. More and more genetic variants resulting in PPGLs are found increasing our understanding and changing our follow-up of these conditions (37). Metastatic PPGLs can appear many years after the initial surgery and curative therapy can then be difficult to achieve (41).

## Primary aldosteronism

Primary aldosteronism (PA) is severely underdiagnosed (42). It has been estimated that 4-14% of all patients with hypertension in primary care have it secondary to primary aldosteronism if properly investigated (43–45). However, the investigations are cumbersome (46, 47), and easier algorithms are urgently needed. Unilateral hypersecretion of aldosterone, usually due to an aldosterone producing adenoma, is seen in approximately half of all patients with PA, with overproduction from both adrenals in the remaining cases, usually due to bilateral idiopathic hyperplasia. The treatment of choice if the patient is operable is adrenalectomy in unilateral PA and mineralocorticoid receptor antagonists (MRAs) for bilateral PA (46). Functional histopathology can improve both histological diagnosis and predict failure after adrenalectomy (48). If PA is left without specific treatment, the risk for cardiovascular diseases, chronic kidney disease and mortality are increased compared to essential hypertension (49). Unilateral adrenalectomy has been considered superior concerning cardiovascular outcomes and quality of life compared to medical treatment in unilateral disease (50, 51). However, the dose of MRA is often suboptimal and an optimal dose may be more equivalent to adrenalectomy in unilateral disease, but this has to be investigated in future studies.

## Conclusion

Adrenal disorders are often misdiagnosed, and the management can be challenging. Some disorders are very rare while others are common but the knowledge about them among physicians and the general population are low. More RCTs and collaborations are required to improve the management and our understanding of adrenal disorders. Hopefully, the Section Adrenal Endocrinology at Frontiers in Endocrinology can be a place where new ideas and collaborations will thrive while the challenges are met.

## Author contributions

The author confirms being the sole contributor of this work and has approved it for publication.
